# Dementia Friendly Communities in England: A scoping study

**DOI:** 10.1002/gps.5123

**Published:** 2019-05-20

**Authors:** Stefanie Buckner, Nicole Darlington, Michael Woodward, Marina Buswell, Elspeth Mathie, Antony Arthur, Louise Lafortune, Anne Killett, Andrea Mayrhofer, John Thurman, Claire Goodman

**Affiliations:** ^1^ Cambridge Institute of Public Health University of Cambridge Cambridge UK; ^2^ Centre for Research in Public Health and Community Care University of Hertfordshire, College Lane Hatfield UK; ^3^ Norwich Research Park University of East Anglia Norwich UK; ^4^ Collaboration for Leadership in Applied Health Research and Care East of England, Douglas House Cambridge UK

**Keywords:** awareness raising, dementia, Dementia Friendly Communities, England, evaluation tool, inclusion, older adults, online data

## Abstract

**Objectives:**

To describe the characteristics of Dementia Friendly Communities (DFCs) across England in order to inform a national evaluation of their impact on the lives of those affected by dementia.

**Methods:**

DFCs in England were identified through online searches and Alzheimer's Society records. A subsample (n = 100) were purposively selected for in‐depth study based on online searches and, where necessary, follow‐up telephone calls. Data collection and analysis were guided by a pilot evaluation tool for DFCs that addressed how DFCs are organised and resourced and how their impact is assessed. The evidence was predominantly qualitative, in addition to some descriptive quantitative information.

**Results:**

Of 284 DFCs identified, 251 were defined by geographical location, while 33 were communities of interest. Among 100 sampled DFCs, 89 had been set up or started activities following policy endorsement of DFCs in 2012. In the resourcing of DFCs, statutory agencies and charities played an important role. Among DFC activities, awareness raising was cited most commonly. There was some evidence of involvement of people living with dementia in organisational and operational aspects of DFCs. Approaches to evaluation varied, with little evidence of findings having effected change.

**Conclusions:**

DFCs are characterised by variation in type, resourcing, and activities. England has policy endorsement and a recognition system for DFCs. These can be important catalysts for initiation and growth. A systematic approach to evaluation is lacking. This would enable DFCs to be consistent in how they demonstrate progress and how they enable people living with dementia to live well.

**Key points**

- This is the first national overview of Dementia Friendly Communities (DFCs). It was carried out in England as one of the few countries that have incorporated DFCs into policy.
- DFCs are characterised by variation in type, resourcing, and activities.
- Policy endorsement was an important driver for the growth of DFCs across the country.
- An agreed approach to evaluation could support DFCs in how they monitor their progress, involve people living with dementia, and agree on criteria for good practice for DFCs in different contexts and at different stages of development.

## 1 INTRODUCTION

Growing recognition in recent years of dementia as an urgent global health issue^1^ has led to an increase in Dementia Friendly Communities (DFCs). While there are many different kinds of DFCs, they share the common goal of ensuring that people affected by dementia (those living with the condition and their supporters and carers) can continue to be active and valued citizens.^2^ Ninety percent of Organisation for Economic Co‐operation and Development (OECD) countries support DFC initiatives.^3^ In individual countries, efforts to create dementia‐friendly environments have been ongoing for some time, such as in Japan,^4^ where initiatives can be traced back to at least 2004. In the United Kingdom,^5^ it was the Prime Minister's Challenge in 2012 that put DFCs on the agenda. England is one of the few countries that has incorporated the creation of DFCs into policy, with targets for the creation of DFCs and a system of recognition linked to standards.^5^, ^6^, ^7^, ^8^, ^9^, ^10^, ^11^

Fundamental to DFCs is the involvement of people living with dementia in all aspects of their organisation and operations.^10^, ^11^ A more contested aspect is the term “dementia friendly” itself. While apparently positive and laudable in its intentions, it has been criticised for advocating charitable kindness towards people living with dementia. What is needed instead, it has been argued, is a rights‐based approach that focuses on the removal of socially imposed barriers and on enablement.^12^ Calls for recognition of the human rights of people living with dementia have been growing louder in recent years.^13^, ^14^, ^15^

There has been growing interest in the concept of DFCs, and a substantial body of research exists.^2^ This ranges from studies on what it means to be a citizen with dementia^16^, ^17^ to evaluations of communities' activities^18^ and evaluations of dementia‐sensitive infrastructure such as transport and the design of public and commercial buildings.^19^, ^20^ Most published evaluations of DFCs were completed within the first few years of the initiatives having been set up. 21, 22, 23

With DFCs now supported by national policy, there is a need to know how they are configured and characterised and how they prioritise activities. This paper presents early findings from the National Evaluation of Dementia Friendly Communities in England (the DemCom Study, January 2017 to June 2019), funded by the Department of Health and Social Care. It provides an overview of DFCs in the country. The research question that informed this work was as follows: What are the characteristics and foci of DFCs in England?

The DemCom Study adopted a broad working definition of DFCs so as to capture the range of possible approaches and encompass groups or organisations that self‐identified as DFCs:

A Dementia Friendly Community can involve a wide range of people, organisations and geographical areas. A DFC recognises that a person with dementia is more than their diagnosis, and that everyone has a role to play in supporting their independence and inclusion.

DemCom has drawn on related work on evaluating the impact of the World Health Organization's (WHO) Age‐Friendly Cities initiative.^24^, ^25^ Together with existing guidance for aspirant DFCs,^9^, ^10^, ^11^ this work^26^, ^27^, ^28^ has helped to identify the characteristics of DFCs examined in this article.

## 2 METHODS

### 2.1 Identification and sampling of DFCs

Identification of DFCs and data collection took place between January and June 2017. Records of communities that had been formally recognised as “working towards being a DFC” by the Alzheimer's Society^11^, ^29^ were obtained from the Society. Formal recognition entails a community successfully demonstrating its commitment to meeting the seven “foundation criteria” for DFCs and monitoring and reporting on its progress towards them.^9^, ^10^ Alzheimer's Society records were complemented by online searches in Google, based on the following search terms: “Dementia Friendly Communit*”; “Dementia Friendly*”; “Dementia Action Alliance”; “Dementia Friends”. In addition, a “Google Alert” that generated notifications of the term “dementia friendly” occurring in news articles was in place.

**Figure 1:**
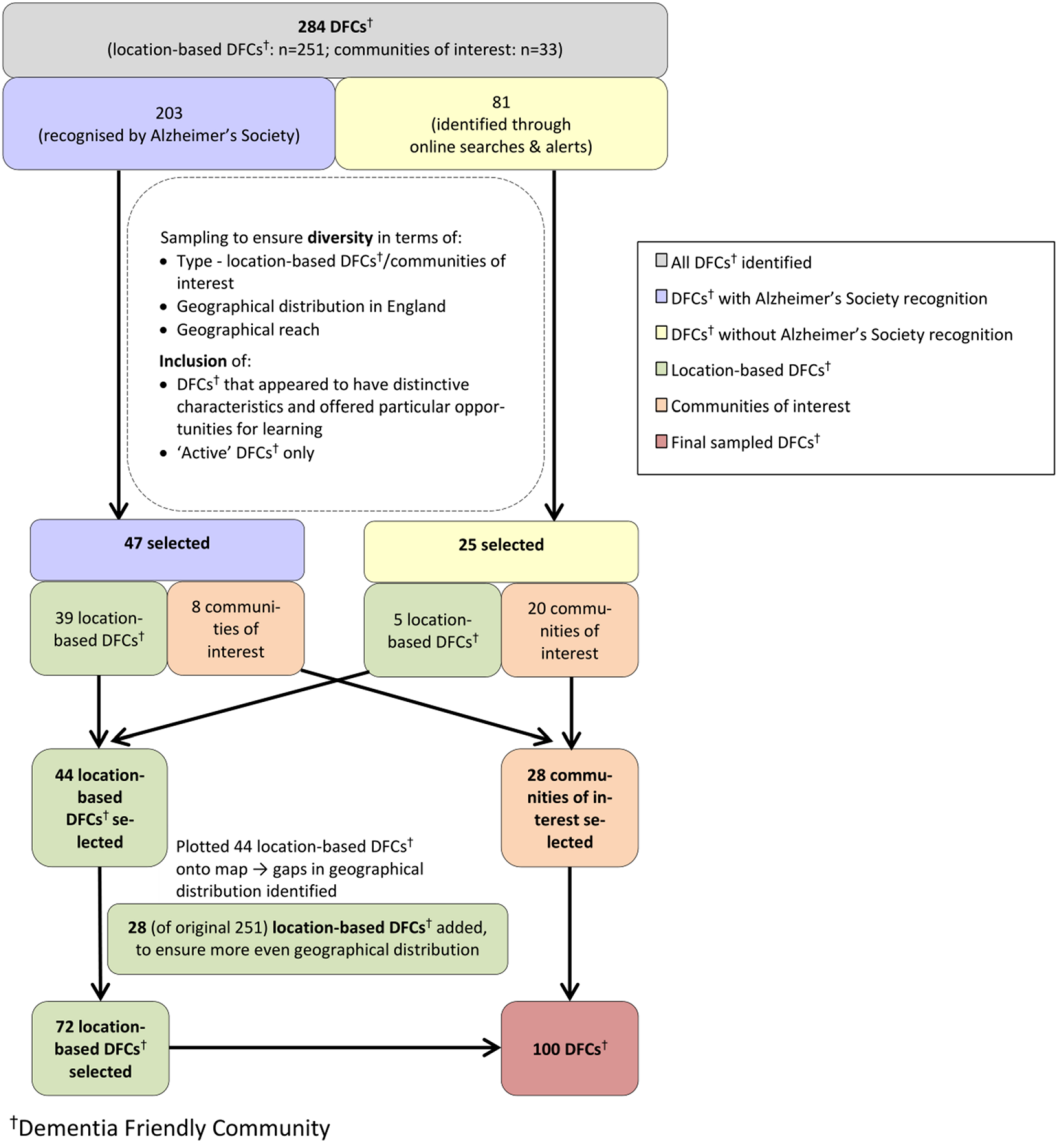
Selection process for 100 sampled Dementia Friendly Communities (DFCs) in England [Colour figure can be viewed at wileyonlinelibrary.com]

Following initial mapping of all DFCs, a selection (n = 100) were examined in depth. These were purposively sampled to reflect the diversity of DFCs by the following: (a) type—DFCs defined by their location (eg, cities and counties) called “location‐based DFCs” and DFCs that are organisations or entities with a specific focus (eg, an airport and a national supermarket chain) summarised as “communities of interest”; (b) geographical distribution across England; and (c) geographical reach/size. Additionally, DFCs were included if the available data indicated characteristics that made them distinctive and offered particular opportunities for learning—for example, an explicit concern with the rights of people living with dementia or attention to particular groups (eg, Black and Minority Ethnic communities). Only “active” DFCs were selected, defined as DFCs where online sources suggested activity in the previous 6 months or whose active status was confirmed in a telephone call. The different steps of the sampling process are outlined in Figure 1.

### 2.2 Data collection and analysis

A multimethod approach to data collection was used. Online searches of DFC and related websites (eg, local government and voluntary sector) were carried out to obtain key information on the 100 sampled DFCs. Stansfield et al^30^ provide a three‐stage framework for systematically identifying online information. Initially, Google was selected for the online searches. Next, the following search terms were applied consecutively: “Dementia Friendly [name]”; “[name] Dementia Friendly Community”; “Dementia Action Alliance [name]”; “Dementia Friends [name].” In a third step, this process was stopped once a minimum of four, and up to seven, online data sources for each identified DFC (including DFC website and reports in local media) had been selected from up to four pages of search results. The aim was to identify sufficient online information to complete a data extraction form and gain a comprehensive picture of a DFC. Where gaps remained and contact details for a DFC were available, up to three attempts at a follow‐up telephone call to a stakeholder (such as a DFC coordinator) were made to obtain further information.

**Figure 2:**
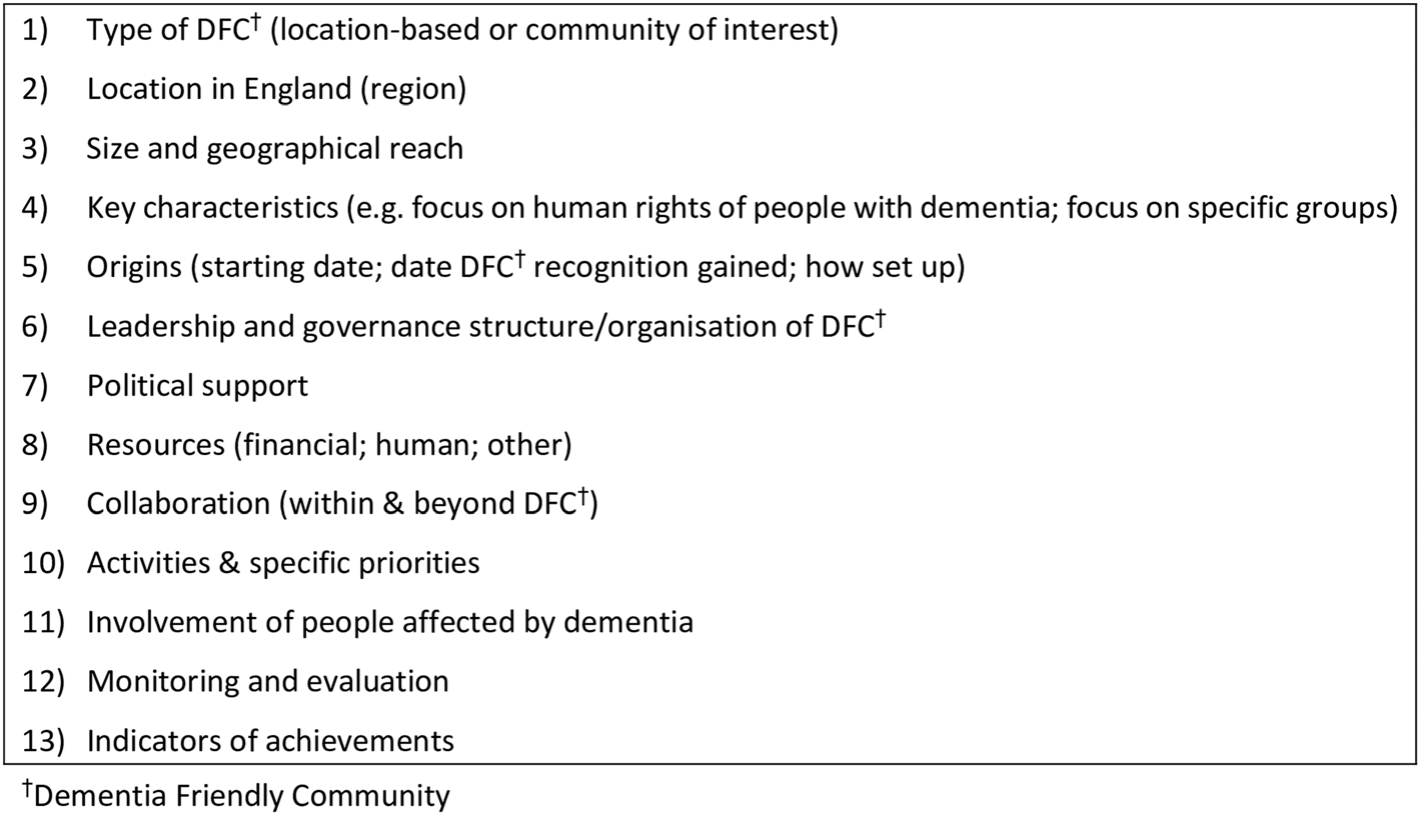
Data extraction form for sampled Dementia Friendly Communities (DFCs)

The data extraction form was based on a preliminary version of an evaluation tool for DFCs being developed as part of DemCom, which identified different thematic areas for which evidence needed to be collected. This had its roots in an evaluation tool developed for Age‐Friendly Cities.^26^ Thematic areas for data extraction included how a DFC was led and governed, what activities it involved, how people affected by dementia (people living with dementia as well as their carers/supporters) were involved in a DFC, and whether and how a DFC's work was monitored and evaluated (Figure 2). From these thematic areas, key characteristics of DFCs were distilled (eg, size and resources; see Sections 3.1 to 3.7).

The area of DFC activities was particularly complex. It required close examination that entailed categorising all DFC activities identified by purpose and intended target group (see Section 3.5).

All authors were involved in data extraction. Double extraction was carried out for 17 DFCs to ensure a systematic and reliable approach. Team discussion resolved how ambiguous data were categorised. Coding and analysis were carried out by four members of the research team using MS Excel (version 2016).

This paper is based solely on information available in the public domain. Contacts who were telephoned were alerted to this, and only publicly available documents were accessed. This phase of the study was assessed as not requiring formal ethical review.

## 3 RESULTS

A total of 284 DFCs were identified across England—the majority (n = 203) from Alzheimer's Society records of communities formally recognised as working towards being a DFC, and 81 from additional sources. Table 1 presents an overview of the characteristics of the 284 DFCs identified and how they are reflected in the 100 sampled DFCs.

**Table 1:** Overview of number of DFCs in England (n = 284) and sampled DFCs (n = 100) according to sampling criteria

Sampling Criteria			n out of 284 DFCs in England	n out of 100 Sampled DFCs
Type of DFC		Location‐based	251	72
		Communities of interest	33	28
Location in England		South West	49	14
		South East	47	11
		London	14	7
		East of England	40	13
		West Midlands	22	10
		East Midlands	14	4
		Yorkshire & Humber	28	10
		North West	34	15
		North East	25	7
		National or N/A	11	9
Geographical reach	DFCs that clearly define their geographical reach	County	15	8
		City	30	18
		Town	123	27
		Village	14	3
	DFCs that have less clear boundaries/align with local administration areas	Unitary Authority	5	5
		Borough	19	4
		District	24	3
		Parish	12	1
		Other (including communities of interest)	42	31
Additional features		Data indicate concern with human rights of people living with dementia	8	8
		Data indicate attention to particular groups (Black and Minority Ethnic; Lesbian Gay Bisexual Transgender)	7	7
		No additional features	269	85
Active status		Yes	204	100
		No	26	0
		Missing data	54	0

Abbreviation: DFC, Dementia Friendly Community.

### 3.1 Online presence of DFCs

The online presence of the 100 sampled DFCs was variable, as were the quality and range of data that could be extracted for them. For some DFCs, fewer than four online sources were available, with available sources ranging from one to 10. Insufficient online information to populate the data extraction sheet resulted in attempted telephone contact with 22 DFCs. This was successful in 13 DFCs, for which additional information was obtained.

### 3.2 Types of DFCs, geographical reach, and size of population served

Of the 100 sampled DFCs, 72 were location based, and 28 were communities of interest. It is a target for 2020 that over 50% of the English population will be living in a DFC.^7^ The number of people living in the location‐based DFCs ranged from 850 in a small parish to 5 300 000 in a county. The majority of the location‐based DFCs covered comparatively large urban areas—more than a third (n = 27) were towns, and a quarter (n = 18) were cities. It is worth noting that there were cases where DFCs overlapped, for example, where a town with DFC status was located within a county DFC.

The 28 communities of interest included housing associations, churches, airports, banks, a supermarket chain, a police constabulary, a fire department, a university, support groups, a dental surgery, and associations with a focus on cultural activities. Precise figures for the population they reached could not be identified. Many were located within location‐based DFCs but appeared to be self‐regulating in their organisation.

### 3.3 Origins, organisation, and ways of working

While DFCs have policy support, their characteristics—how they are organised, their priorities, and the ways in which they work—reflect by whom they have been led and how long they have been in place. The time when the sampled DFCs had been established, or when their work on dementia had started, ranged from almost two decades ago (1998, in one case) to the previous year. The DFC whose activities date back to 1998 had developed from an organisation to support carers and people living with dementia among the African/Caribbean community. The vast majority of the DFCs (n = 89) had been set up or started their activities since 2012, the year in which DFCs were endorsed by policy through the first UK Prime Minister's Challenge on Dementia.^5^

In 45 of the 72 location‐based DFCs and 18 of the 28 communities of interest, it was possible to identify key aspects of their history that had shaped their evolution. For 21 of the location‐based DFCs, local needs assessments, dementia being a local government priority, and community initiatives for people living with dementia had formed the basis for becoming a DFC. In the case of the communities of interest, joining an already growing movement such as a local Dementia Action Alliance^31^ and acting on Alzheimer's Society guidance on dementia friendliness played an important role. A further factor was a recognition by the communities of interest of the responsibility they had to people affected by dementia who used their services (eg, church members and shoppers).

In over half of the sampled DFCs (n = 53), collaborations between diverse agencies and individuals had shaped how the DFC had started and was being promoted. Regarding the 72 location‐based DFCs, public sector organisations such as councils/local authorities, clinical commissioning groups (CCGs), and emergency services were involved in the creation of almost half (n = 34) of them, often in partnership with each other and local charities. Volunteers were reported as having had a role in initiating just under a third of them (n = 21).

Linked to the support from local government, there was some evidence of political endorsement of DFCs. Of the 72 location‐based DFCs, 11 noted the backing and practical involvement of elected government representatives (members of Parliament). The data also indicated political engagement, for example, in the form of locally elected officials (mayors and councillors) participating in DFC‐related events and activities. In contrast, in a small number of settings (n = 3), there was evidence of attempts to keep politics separate, emphasising locally grown leadership and involvement. In the case of the 28 communities of interest, the level of political support was not identified.

### 3.4 Resources

DFCs had varying—and often multiple—sources of income. For the majority of the DFCs studied (n = 54), it was unclear how their activities were supported or if there was long‐term funding. Where it was reported, funds available to DFCs ranged from £200 from a fundraising event to £1 million of government funding for improvements to care homes badged as making the borough more dementia friendly. Almost a third of DFCs (n = 29) had received grants, commonly from their local authority, but also from CCGs and voluntary sector organisations. Larger grants were funding improvements to infrastructure. One city council, for example, had allocated £250 000 to making customer facing council buildings dementia friendly. Fundraising and/or donations were further sources of income identified in a substantial number of cases (n = 15).

Almost half of the sampled DFCs (n = 48) reported access to salaried staff with support from local government, health care commissioners, charities, and local partnerships. It was unclear whether these roles had an exclusive DFC focus or whether staff were employed to deliver on specific projects (eg, promoting dementia‐friendly businesses and transport). In 35 DFCs, more than one salaried position relevant to the DFC initiative was reported, but only eight DFCs differentiated between full‐time and part‐time employees. Volunteer input was referenced in just over a fifth of cases (n = 22).

Three DFCs reported in‐kind support for dementia‐related activities, including free and subsidised use of facilities such as meeting rooms, and administrative support from a charity.

### 3.5 Work on dementia—focus and activities

There is a clear policy imperative for DFCs to address the stigma of living with dementia.^32^, ^33^ Among the DFCs, there was a strong sense of a commitment to promoting awareness of the needs of people living with dementia and finding ways of supporting participation in everyday activities. In the 72 location‐based DFCs, a total of 269 activities were reported. The focus of half (n = 132) of these was awareness raising in the wider community, with sessions to create Dementia Friends (community members who have gained a better understanding of living with dementia^34^) (n = 45) and Dementia Friends Champions (volunteers helping others to learn about living with dementia and become Dementia Friends^35^) (n = 11). Activities that created social media presence, information leaflets, and individual events were also widely reported. Awareness raising was also the most common activity among the 28 communities of interest, with 20 of them engaging in relevant activities, such as running Dementia Friends sessions for staff or the wider community.

Some DFCs offered a range of activities and services for people affected by dementia. Of the 269 activities in location‐based DFCs, a quarter (n = 69) were identified as attracting/“bringing in” users to venues that had been designated for a dementia‐related purpose (eg, memory cafés). Slightly fewer (n = 59) offered activities in which users had opportunities to be involved as part of the wider community (eg, in leisure and sports). There were initiatives and services that were designed exclusively for people living with dementia (eg, reminiscence groups) or for their supporters (eg, carer support groups). In some cases, DFCs conflated the need to provide practical support and services for people affected by dementia with their role as promoters of community engagement and social inclusion.

Despite policy directives to promote the rights of people living with dementia as citizens and to challenge environments and attitudes that disable and stigmatise them,^13^, ^14^ only two DFCs made explicit reference to a rights‐based approach informing their work.

### 3.6 Involvement of people affected by dementia

The involvement of people living with dementia and their supporters and carers in the setting up, running, and monitoring of DFCs indicates their recognition as experts by experience or active agents able to direct, contribute, and participate.^17^, ^36^ There was evidence of involvement for a fifth (n = 20) of the sampled DFCs. This included people living with dementia acting as chairs of meetings, contributing to steering groups, and carrying out audits of how dementia friendly the local environment was. For a slightly larger group of DFCs (n = 27), involvement could be inferred from references to consulting people living with dementia on DFC priorities and a narrative on the importance of involvement. Statements emphasising the fact that people affected by dementia were contributing to a DFC were common. In over half of the DFCs (n = 53), the extent and nature of involvement was not described. The ways in which the contributions of people affected by dementia shaped DFC strategy and activity also remained unclear.

### 3.7 Monitoring and evaluation

In a third of the DFCs studied (n = 33), formal monitoring and evaluation were mentioned, defined as efforts to assess performance and/or progress within the DFCs. This included evaluations of specific projects (eg, setting up a dementia‐friendly high street). More than half of DFCs (n = 55) provided updates on what they had achieved. Commonly used indicators were numbers of Dementia Friends and Dementia Friends Champions; number of dementia‐friendly businesses and dementia‐related activities; achieving DFC recognition by the Alzheimer's Society; and extent of membership of a Dementia Action Alliance or comparable group. In two of the DFCs, monitoring and evaluation had been planned but not progressed beyond an exploratory stage of what data could be collected. In three DFCs, there were accounts of how findings had led to documented changes, for example, activities being altered based on feedback from people affected by dementia. In one further DFC, a self‐assessment of progress made against recommended actions for becoming dementia friendly had been used for review and planning. Outcome measures such as number of people affected by dementia known to a DFC, evidence of barriers to participation being removed, and examples of changes in service provision (eg, signage; transport; and use of culture and leisure facilities by people living with dementia) were either not stated or implied.

## 4 DISCUSSION

England is one of the few nations in the world to have incorporated the creation of DFCs into policy.^3^ DFCs are spread across the country. The presence of a DFC is associated with the number of dementia cases (known and unknown) but not with the proportion of the population with dementia (prevalence).^37^ This scoping of DFCs has provided the first national overview of DFCs in terms of their key characteristics—how they are organised, how they involve people affected by dementia, what the focus of their work is, and how they measure impact.

The findings reported here are similar to those presenting the experience of Japan, where government endorsement coupled with support for implementation through campaigns and policies resulted in a proliferation of DFCs.^4^, ^38^ Statutory agencies, and especially councils/local government, working in partnership with different bodies and through local collaborations such as Dementia Action Alliances have played a central role in the setting up, managing, and resourcing of the DFCs reviewed.

The main emphasis of the reviewed DFCs was on awareness raising. There was evidence of the ongoing involvement of people living with dementia in DFCs in advisory, operational, and strategic capacities. However, the centrality of citizen involvement was not as clearly articulated as in the literature.^10^, ^11^, ^39^, ^40^, ^41^, ^42^, ^43^, ^44^ This implies the need for further research that can provide a more detailed picture of both the extent and the nature of involvement.

DFCs have been promoted as a potentially cost‐effective model for supporting people affected by dementia. Attention has been paid to the economic aspects of DFCs.^45^, ^46^ An analysis by Green and Lakey^40^ indicates the cost saving potential of DFCs where they enable people living with dementia to live in the community for longer, thus delaying their admission to institutional care. The majority of the DFCs studied did not report how they were resourced. The involvement of local government, as well as health care organisations and charities, can improve access to funding. However, the ad hoc and often short‐term nature of funding that was reported raises questions about what resources are required to enable people affected by dementia to participate meaningfully in their local communities. Investment of people and time in raising awareness was the favoured approach, and it may be that increasing acceptance is a key enabler for people affected by dementia.^47^ Given the absence of a publicly available strategy, it is not possible to know how DFCs use resources, or what resources they would need, to achieve objectives.

DFCs have the potential to contribute over time to improving the quality of life of people affected by dementia. Monitoring and evaluation were underdeveloped in the DFCs reviewed. Pursued from the outset, they could help communities to focus on what enables people affected by dementia to live well, and evaluation guidance for DFCs is available.^48^ However, an evidence‐based evaluation tool able to support internal review, planning, and national comparisons would encourage a more systematic and strategic approach.

In a critique of how businesses become dementia friendly, Connell et al^49^ draw on a civil society perspective^50^ to explain the stages that organisations go through when engaging (or not) with people living with dementia. Starting with denial that dementia is a problem that needs to be addressed, they ultimately achieve normalisation where the needs of people living with dementia are seamlessly integrated without being a niche activity or market. Evaluations of the Bradford and York DFCs^43^, ^44^ make similar points. They suggest that enabling people living with dementia to access mainstream services is where DFCs should start whilst accepting that dementia‐specific activities can sit alongside accessible mainstream services.

… it should be a starting point that people with dementia should be able to access mainstream services and resources in a Dementia Friendly Community alongside everybody else – this is, in essence, the core meaning of the term. At the same time, people with dementia should also of course have the right to choose to engage in specific ‘dementia‐only’ activities as well.,^44^ ^p24^

The findings suggest that access to services, and concern with the rights of people living with dementia, were not the starting points for most DFCs. This implies a need to observe further how DFCs are responding to growing calls for recognition of the rights of people living with dementia to identify if action is required. A focus on awareness raising arguably signalled that most DFCs were concentrating on building structures of support and community responsiveness. Evidence of tangible progress on these issues, however, was difficult to find. A few DFCs also offered dementia‐specific services. These, some would argue, could have the unintended consequence of further separating people living with dementia from their community.^15^ While policy support and a system for formal recognition of DFC status have provided an impetus for DFCs to be set up and/or start their activities in England, they have not led to a consistent national approach.

This work and the related review of how DFCs have developed internationally^47^ demonstrate the importance of an evaluation framework that enables a nascent DFC to identify from the outset relevant progress and impact indicators and a plan for measuring these.

The research has limitations. It provides a snapshot of DFCs that is constrained by the availability of online data, specifically where no follow‐up telephone calls were made. There is a risk of relevant information being underreported. For example, the role of volunteers or of in‐kind support in the DFCs may be greater than the findings suggest. There is a potential selection bias in that the 100 DFCs were purposively selected, although the consistency of their characteristics would suggest they are not atypical. Online resources are only as useful as they are up‐to‐date. As the means by which people affected by dementia can locate information and support, they are a proxy measure of the visibility and accessibility of a DFC. The fact that 26 DFCs of the original 284 DFCs did not appear to be active is worth noting.

## 5 CONCLUSION

The findings provide the first national overview of what DFCs are and how they operate. DFCs in England are characterised by variation on key features including type, resourcing, and activities. In order to arrive at an in‐depth understanding of how DFCs can enable people affected by dementia to live well, there is a need to move beyond description to establish the criteria for “good” DFCs in different settings and for different populations. These findings provide a reference point for future work and monitoring of change over time. They have informed the next phase of the DemCom study, in which selected DFCs are being examined in detail, and where an evaluation tool for DFCs is being developed.

## DATA AVAILABILITY STATEMENT

The data that support the findings of this study are available from the corresponding author upon reasonable request.

## CONFLICT OF INTEREST

None declared.
